# Copper (II) Ions Activate Ligand-Independent Receptor Tyrosine Kinase (RTK) Signaling Pathway

**DOI:** 10.1155/2019/4158415

**Published:** 2019-05-14

**Authors:** Fang He, Cong Chang, Bowen Liu, Zhu Li, Hao Li, Na Cai, Hong-Hui Wang

**Affiliations:** ^1^Institute of Nanotechnology and Tissue Engineering, College of Biology, Hunan University, Changsha, 410082, China; ^2^CellWay Bio, Changsha, 410000, China; ^3^State Key Laboratory of Chemo/Bio-Sensing and Chemometrics, College of Chemistry and Chemical Engineering, Hunan University, Changsha, 410082, China

## Abstract

Receptor tyrosine kinase (RTK) is activated by its natural ligand, mediating multiple essential biological processes. Copper (II) ions are bioactive ions and are crucial in the regulation of cell signaling pathway. However, the crosstalk between copper (II) ions and RTK-mediated cellular signaling remains unclear. Herein, we reported the effect of copper (II) ions on the ligand-independent RTK cellular signaling pathway. Our results indicate that both EGFR and MET signaling were activated by copper (II) in the absence of the corresponding ligands, EGF and HGF, respectively. Consequently, copper (II) ions initiate two RTK-mediated downstream signal transductions, including AKT and ERK. Moreover, copper (II) significantly increased proliferation and cellular migration. Our study proposes a novel role of copper in RTK-mediated signaling for growth factor-independent cancer cell proliferation and migration, implying that targeting both the copper (II) and growth factor in tumor microenvironments may be necessary for cancer treatment.

## 1. Introduction

Receptor tyrosine kinase (RTK) is the most abundant type of enzyme-linked receptor, and it is both a receptor and an enzyme that can bind to the ligand and phosphorylate tyrosine residues of target proteins. RTK is a class of single transmembrane receptors with endogenous protein tyrosine kinase activity in cell receptors [1]. So far, more than 50 RTKs have been identified, including hepatocyte growth factor receptor (MET), epidermal growth factor receptor (EGFR), vascular endothelial growth factor receptor (VEGFR), platelet-derived growth factor receptor (PDGFR), and fibroblast growth factor receptor (FGFR) [2, 3]. All members of RTK have similar protein structures: extracellular ligand binding domain, single transmembrane helical domain, near-membrane regulatory domain, a tyrosine kinase domain, and carboxyl-terminal region. Most ligands that specifically activate RTK are soluble secretory proteins, called growth factors. When the growth factor binds to the extracellular domain of RTKs, the receptor is induced to dimer by ligand, and the protein conformation changes to enhance the kinase activity of RTK [4]. The RTK signaling pathway is strictly regulated by various positive feedback loops [5]. The RTK signaling pathway regulates cell proliferation, and differentiation promotes cell survival and regulates and corrects cell metabolism [6]. At present, the RTK signaling pathway has become the primary target in tumor therapy such as breast cancer, prostate cancer, glioblastoma, pancreatic cancer, and lung cancer [7]. EGFR (epidermal growth factor receptor) is a receptor for cell proliferation and signal transduction in epithelial growth factor (EGF). EGFR dimerization activates its intracellular kinase pathway and directs downstream phosphorylation, including the MAPK, AKT, and JNK pathways, to induce cell proliferation [8, 9]. MET (hepatocyte growth factor receptor, HGFR) plays a vital role in cell morphology, proliferation, differentiation, migration, and survival. The signal transduction pathway, which is of great significance, is shown to be active in many tumors. MET-HGF/SF is a potential therapeutic target [10]. AKT (a.k.a. protein kinase B, PKB) is a protein serine/threonine kinase activated by inositol phosphate recruitment to the plasma membrane, which plays a significant role in cell survival and apoptosis [11]. ERK (extracellular regulated protein kinases) refers to extracellular regulated protein kinases, including ERK1 and ERK2, which are the key to transmitting signals from surface receptors to the nucleus. ERK is engaged in many biological reactions such as apoptosis, cell carcinogenesis, cell proliferation and differentiation, cell morphology maintenance, and cytoskeleton construction [12].

Copper is a necessary metal in biology and is widely found in prokaryotes, fungi, mammals, plants, and humans [13]. The vital role of copper in a series of critical physiological processes is increasingly demonstrated in various research fields including wound healing, angiogenesis, protection of reactive oxygen species, synthesis of neurotransmitters, regulation of normal cells, and tumor growth [14]. For example, increased copper content in tumor microenvironments is directly related to the progression of many malignant tumors. It has been reported that CD 147 autocorrelation induced by copper targeting is a new tumor therapy strategy [15]. Copper has been involved in the regulation of the immune response and plays an essential role in regulating gene expression and the maturation of fine hypertrophic cells [16]. Copper has excellent antibacterial properties, and it is not easy for bacterial resistance to develop in response to it. Copper ions can slow down inflammation and have high potential applications in the pharmaceutical, health, food industry, agricultural, and other sectors [17]. The role that copper ions play in inflammatory reactions, oxidation pressures, and microbial environments should not be underestimated. Wound healing is related to hemostasis, inflammation, proliferation, scabbing, and so on [18, 19]. Copper is also known to promote angiogenesis and the development of new blood vessels that are essential to feeding rapidly growing and dividing cells, including rampantly dividing cancer cells. Indeed, copper stimulates the formation of vascular and mature factors such as vascular endothelial growth factor (VEGF) [20]. On the other hand, copper exists in either a reduced (Cu^+^) state or an oxidized copper (II) (Cu2^+^) state in structure and catalysis [21]. Although copper is involved in many aspects of cell signal transduction and cellular functions, the mechanisms of this activity remain less well understood.

Herein, we hypothesized that copper (II) ions promote cell proliferation via an RTK-mediated signaling pathway. Therefore, we investigated the effect of copper (II) on the RTK-mediated cellular signaling pathway. The current study aimed at finding out the influences of copper (II) ions on RTK-mediated cellular signaling pathway and cellular responses including proliferation and wound healing, providing useful data for further study on the mechanism of copper (II) ions' actions in cell behaviors.

## 2. Materials and Methods

All experiments in this study were performed in the Institute of Nanotechnology and Tissue Engineering, College of Biology, Hunan University.

### 2.1. Reagents and Instruments

#### 2.1.1. Reagents

Copper dichloride (CuCl_2_) was purchased from Sangon Biotech (Shang Hai, China). 3-(4,5-Dimethyl-2-thiazolyl)-2,5-diphenyl-2-H-tetrazolium bromide (CCK-8) was purchased from Sigma-Aldrich (China). The primary antibody for phospho-EGFR (Y1068, #4064) and phospho-AKT (S473, #4007) was obtained from Bioworld Technology. The primary antibody for phospho-MET (Y1234/Y1235, #3077), total-MET (#8198), and phospho-ERK (T202/Y204, #3510) was obtained from Cell Signaling Technology. The secondary antibody (goat anti-rabbit IgG(H&L)-HRP, goat anti-mouse IgG(H&L)-HRP) was obtained from Invitrogen. The *α*-tubulin primary antibody was purchased from Cellway Biological Co., Ltd. Recombinant Human Hepatocyte Growth Factor (hHGF) and Recombinant Human Epidermal growth factor (hEGF) were obtained from Peprotech. Forenitib, a MET inhibitor, was purchased from Selleck. The nitrocellulose membrane was obtained from Merck Millipore (Germany). RPMI1640, DMEM medium was purchased from neuronbc (Beijing, China); fetal bovine serum (FBS) was purchased from Biological Industries USA. Penicillin-Streptomycin (100X), 0.25% Trypsin-EDTA (1X), and ECL substrate solution were purchased from NCM Biotech (Suzhou, China).

#### 2.1.2. Instruments

Electrophoresis apparatus was purchased from Beijing Liuyi Co., Ltd. The transmembrane instrument was purchased from Biotool. Western blot images were acquired on a chemiluminescence imaging system (MicroChemi4.2). Microporous plate detector was purchased from PerkinElmer, Inc. The inverted fluorescence microscope with no eyepiece was purchased from AMG Co., Ltd. (EVOS f1, America).

### 2.2. Cell Culture

All cells were cultured in 5% CO_2_ in an incubator (Thermo Fisher) at 37°C. A549 cells and DU145 cells were cultured in RPMI1640 with 10% fetal bovine serum and 1% penicillin and streptomycin.

### 2.3. Preparation of Cell Lysates

Cells were seeded in 35 mm dishes. When the cells reached 80% confluence, they were starved for 24 h in 1640 supplemented with 0.2 % FBS. After the starvation, the medium was changed and incubated with different concentration of copper ion for 12 min in the incubator. Then the dishes were put on the ice to stop the stimulation and washed twice by precooling PBS and then lysed with lysis buffer (RIPA buffer with 1% phosphatase inhibitors and protease inhibitor). The cell lysates were centrifuged at 14000 rcf for 10 min; then retain the supernatant, saved in the -20°C before use.

### 2.4. Western Blot Assay

The cell lysates were separated by 8% SDS-PAGE electrophoresis and then transferred to nitrocellulose membrane by semidry electrophoretic transfer unit for 10 min. After blocking with 5 % BSA-PBST (1×PBS with 0.1% Tween-20) solution for 1 h, the membrane reacted with primary antibody (1:1000 dilution) overnight in 4°C and secondary antibody (1:5000 dilution) for 1 h in room temperature. Before imaging, the membranes were reacted with ECL substrate solution (NCM Biotech Co., Ltd). Chemiluminescent images were obtained using Bio-Imaging Systems (MicroChemi4.2), and the density of bands was quantified using Image Studio Lite software (Ver 3.1, Li-Cor).

### 2.5. Cell Viability Assay

The A549 and DU145 (5.0 × 10^2^ cells) were seeded at 96-well plate for 24 h. Then, the medium was removed, and the cells were pretreated with copper (II) ions at various concentrations of 0 *μ*M, 5 *μ*M, 10 *μ*M, 20 *μ*M, 50 *μ*M, and 100 *μ*M. Next, the cells were incubated under 5% CO_2_ in a humidified incubator at 37°C. After 2 days, cell viability was evaluated using CCK-8 according to the manufacturer's instruction. CCK8 solution (10 *μ*l) was added to each well, and the mixtures were incubated for 2 h at 37°C. Absorbance was then measured using a plate detector at 450 nm.

### 2.6. Wound Scratch Assay

Firstly, A549 cells were seeded in a 12-well plate at a density that they should reach ~70-80% confluence as a monolayer after 24h of growth. Do not change the medium, and wounds were then scratched in each cell monolayer using a sterile 1 ml pipette tip. After scratching, gently wash the well twice with medium to remove the detached cells. Then, cells were further cultured with 50 *μ*M copper (II) in the 1640 medium with 0.2 % FBS. The cell motility was measured at 24 h by an inverted microscope. The rate of wound healing was related to the ability of cell migration and cell proliferation.

### 2.7. Statistical Analysis

Statistical analyses were performed using GraphPad Prism 5 software (GraphPad Software, Inc., San Diego, CA). All data are presented as means ± SD, and Student's non-paired t-test was used for statistical analyses. An overall variation among the different groups was analyzed by One-way ANOVA statistical analyses. The asterisk marks significant differences (*∗*P<0.05, *∗∗*P<0.001).

## 3. Results

### 3.1. Copper (II) Promotes Ligand-Independent Activation of RTK

**Figure 1:**
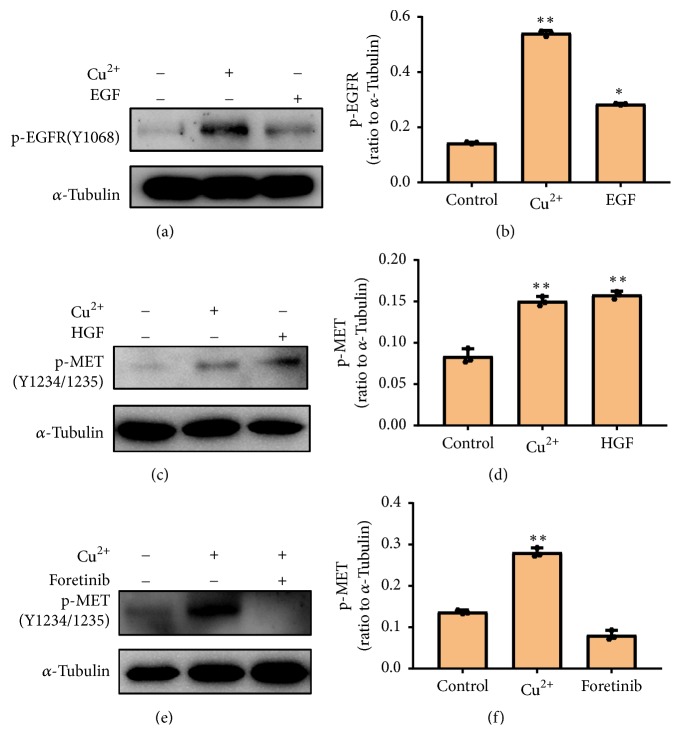
*Copper (II) promotes RTK activation analogous to the ligand-activated pathway*. (a) DU145 cells serum-starved for 24 hours were stimulated with 50 *μ*M copper (II) ion and 99 ng/ml EGF for 10 min, respectively. Copper (II) ion and growth factor on EGFR activation were evaluated by western blotting. (b) The phosphorylation level of EGFR in each experiment was quantified and analyzed. Data are represented as means ± S.D. of triplicate experiments (*∗*P<0.05, *∗∗*P<0.001). (c) A549 cells were serum-starved for 24 hours and stimulated with 50 *μ*M copper (II) ion and 50 ng/ml HGF for 10 min, respectively. (d) The phosphorylation level of MET in each experiment was quantified and analyzed. Data are represented as mean ± S.D. of triplicate experiments (*∗*P<0.05, *∗∗*P<0.001). (e) A549 cells were serum-starved for 24 hours and pretreated without or with foretinib (100 nM) for 2 h. Then the cells were stimulated with 50 *μ*M copper (II) ion for 10 min and subjected to western blotting analysis. (f) The phosphorylation level of MET in each experiment was quantified and analyzed. Data are represented as mean ± S.D. of triplicate experiments (*∗*P<0.05, *∗∗*P<0.001).

**Figure 2:**
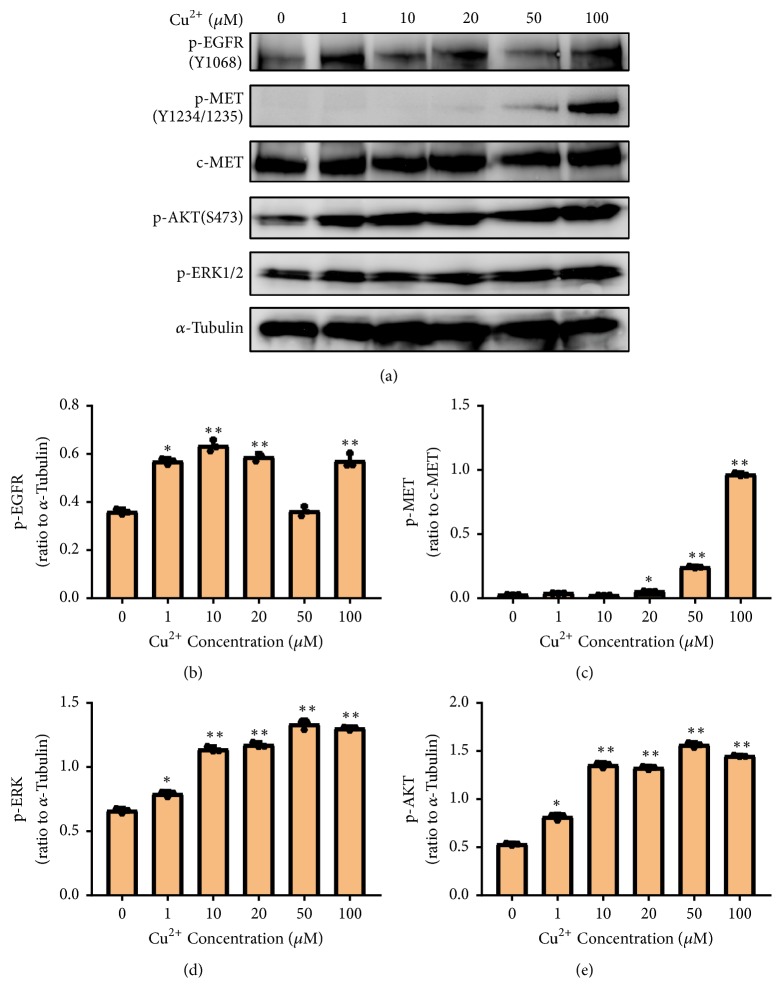
*Copper (II) induced the RTK-mediated cellular signal pathway in a concentration-dependent manner*. (a) A549 cells were treated with different concentrations of copper (II) ion for 10 min and the phosphorylation of EGFR, MET, AKT, and ERK was examined using western blotting. The phosphorylation level of EGFR (b), MET (c), ERK (d), and AKT (e) in each experiment was quantified and analyzed. Data are represented as mean ± S.D. of triplicate experiments (*∗*P<0.05, *∗∗*P<0.001).

**Figure 3:**
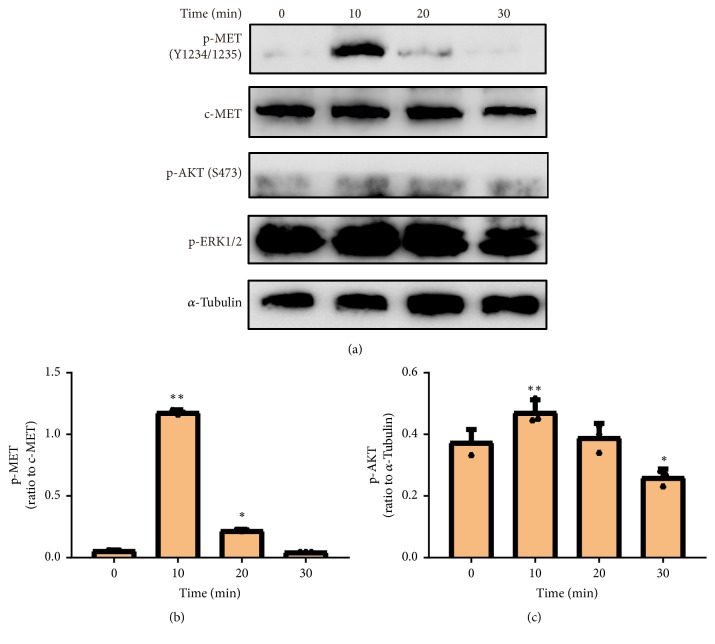
*Copper (II) ion stimulated the RTK-mediated signal pathway time-dependently*. (a) The A549 cells were serum-starved and treated with 100 *μ*M copper (II) for 0, 10, 20, and 30 min, respectively. The phosphorylation of MET, AKT, and ERK1/2 was examined using western blot analysis. The phosphorylation level of MET (b) and AKT (c) in each experiment was quantified and analyzed. Data are represented as mean ± S.D. of triplicate experiments (*∗*P<0.05, *∗∗*P<0.001).

RTK-mediated cellular signal pathway plays an essential role in the human body [1]. In order to explore the effect of copper (II) on RTK-mediated cellular signal pathway, we selected human lung cancer cell A549 and human prostate cancer cell DU145. We firstly investigated the effects of copper (II) on RTK phosphorylation and growth factors (HGF or EGF). The results showed that copper (II) significantly promoted the phosphorylation of EGFR in DU145 cells (Figures 1(a) and 1(b)). Similarly, copper (II) induced the phosphorylation of MET in A549 cells (Figures 1(c) and 1(d)). Both results demonstrated that RTK was able to be activated by copper (II) ions. To investigate whether the kinase activity plays a role in copper (II)-induced RTK activation, foretinib, a potent inhibitor for MET, was utilized, and the result showed that the pretreatment of foretinib significantly inhibited the copper (II)-induced phosphorylation of MET in A549 cells (Figures 1(e) and 1(f)). In conclusion, these data indicated that copper (II)-promoted RTK activation is dependent on RTK-dimerization and autophosphorylation (Figure 2(a)). Both the phosphorylation of EGFR and that of MET were upregulated when the concentration of copper (II) was increased to 100 *μ*M (Figures 2(b) and 2(c)). Interestingly, the phosphorylation of EGFR was significantly enhanced in the presence of 100 *μ*M copper (II), suggesting the RTK is dose-dependently activated by the treatment of copper (II). We further characterized the time course of the copper (II)-promoted RTK signal pathway to determine the optimal time for copper (II) to stimulate the RTK-mediated cellular signal pathway. Based on the time-dependent activation of MET by copper (II), the best stimulation time for copper (II) is 10 min (Figures 3(a) and 3(b)).

### 3.2. Copper (II) Triggers RTK-Mediated Downstream Signal Pathways

We evaluated the RTK-mediated downstream signaling pathways including Ras/mitogen-activated protein kinase (Ras/MAPK) and phosphoinositide 3-kinase/protein kinase B (PI3K/AKT). The A549 cells were cultured in the different concentrations of copper (II) for 10 min and to explore their effect on AKT and ERK phosphorylation. The phosphorylation of both ERK and AKT was remarkably upregulated with the increase of copper (II) concentration (Figures 2(d) and 2(e)). We further characterized the time course of copper (II)-activated RTK signaling. The A549 cells were cultured in 100 *μ*M of copper (II) for 0, 10, 20, and 30 min, respectively, and the phosphorylation level of AKT and ERK1/2 in DU145 cell lysates was measured by western blotting analysis. The data demonstrated that the phosphorylation level of ERK1/2 at T202/Y204 residues was significantly increased in the presence of copper (II) compared with that in the control cells (Figure 3(a)). On the other hand, PI3K phosphorylates and activates AKT, contributing to migratory cell behavior [11]. We observed robust time-dependent activation of AKT by copper (II) (Figure 3(c)). In summary, both EGFR and MET signaling were activated upon copper (II) ion stimulation. When the concentration of copper (II) ion increased to 50 *μ*M, the elevated phosphorylation of MET, AKT, and ERK signals was observed as the copper (II) concentration increased, suggesting that the RTK-mediated downstream signaling pathways were promoted by copper (II) in a time-dependent manner.

### 3.3. Copper (II) Promotes Cell Migration and Proliferation

**Figure 4:**
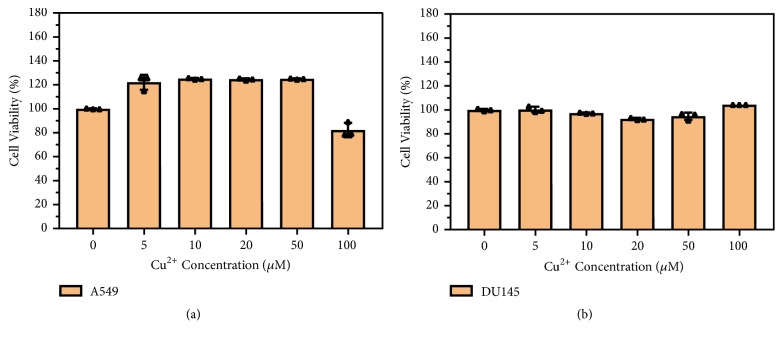
*Copper (II) ion exhibited minimal cytotoxicity on A549 and DU145 cells*. A549 (a) and DU145 (b) cells are incubated with different concentrations of copper (II) ions for 48 h. The cell viability in each experiment was determined using CCK8 assay. Data are represented as mean ± S.D. of triplicate experiments.

**Figure 5:**
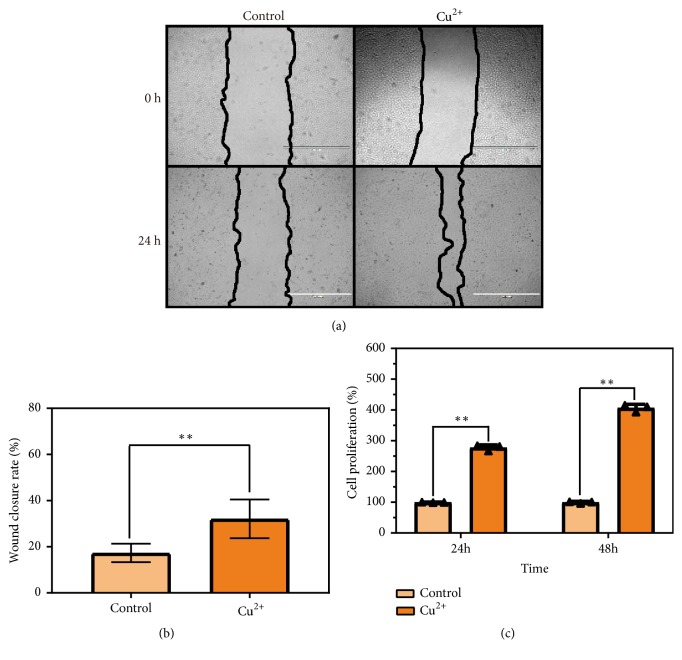
*Copper (II) promoted wound healing and proliferation*. (a) Copper (II) ion enhanced wound healing. The A549 cells were treated with or without copper (II) ion (50 *μ*M), and the wound-closure events were captured by a light microscope. The images were taken at 0 and 24 h. The black lines indicate boundaries between cells in the monolayer and the scratched areas uncovered by cells. Scale bar: 1000 *μ*m. (b) Relative wound-closure rate was measured and analyzed. Data are presented as means ± S.D. (n = 5) (*∗∗*P<0.001). (c) A549 cells were treated with 50 *μ*M copper (II) and the relative cell proliferation at 24 h and 48 h was determined using CCK8 assay. Data are presented as means ± S.D. (n = 5) (*∗∗*P<0.001).

To study the impacts of copper (II) ion on cell viability, A549 and DU145 cells were exposed to copper (II) at different concentrations for analysis. The A549 cells were exposed to 0 *μ*M, 5 *μ*M, 10 *μ*M, 20 *μ*M, 50 *μ*M, and 100 *μ*M copper (II) for 48 h resulting in 100.0 ± 0.3%, 122.1 ± 6.2%, 125.1 ± 0.6%, 124.7 ± 0.8%, 125.0 ± 0.4%, and 82.3 ± 6.0% survival rates, respectively (Figure 4(a)). The survival rates of DU145 cells treated with the different concentrations of copper ion were 99.98 ± 0.9%, 100.3 ± 2.4%, 97.3 ± 0.5%, 92.4 ± 0.9%, 94.7 ± 3.0%, and 104.3 ± 0.0%, respectively (Figure 4(b)). There was no remarkable change in the cell viability after exposure with up to 50 *μ*M copper (II). Next, we used the scratch wound assay to mimic the wound healing in vitro and studied cell migration upon the stimulation of copper (II). We investigated the effect of copper (II) on wound-closure events after making an artificial wound in the monolayer of A549 cells (Figure 5(a)). The treatment of copper (II) ion significantly increased the wound-closure rates (34.7%) of A549 cells (Figure 5(b)), indicating that copper (II) enhanced the cancer cell migration. Moreover, copper (II) ion remarkably promoted cell proliferation (Figure 5(c)). Taken together, copper (II) ion enhanced the cell functions including proliferation and migration via the ligand-independent RTK signaling pathway.

## 4. Discussion

In the present study, we validated that copper could initiate the RTK-mediated signaling pathway and cell functions analogues to the natural ligand biological effect. Interestingly, copper ions used in the treatment of diseases are not suitable for clinical use because of their toxicity, irritability, and absorbability. However, the chelation of the copper ions can reduce their toxicity and irritation and facilitate the cells to absorb copper ions for biological functions [22]. A previous study identified that copper chelate inhibits vascular injury response and promotes angiogenesis for tissue repair [23]. In our experiments, we have titrated the optimal concentration of copper (II) for minimal toxicity to cells and demonstrated that the cells survive and proliferate at the concentration up to 100 *μ*M.

On the other hand, in this experiment, we mainly discussed the effect of divalent copper (II) on the cell signaling pathway and cellular responses. It has been reported that the monovalent copper ion is unstable in the solution, and it is proved that the bivalent copper ion acts on the cell [15]. We determined the optimal time and concentration of the response of the RTK signaling pathway upon copper (II) stimulation. We demonstrated that copper (II) stimulated cells with enhanced phosphorylation levels of RTK (EGFR and MET). The potent inhibitor of MET abolished the effect of copper (II) on phosphorylated-MET, suggesting copper (II) might promote the dimerization-mediated autophosphorylation of RTKs. However, the detailed mechanism should be further investigated. As the critical downstream signaling events, the phosphorylation of ERK and AKT was significantly elevated in the presence of copper (II) in both time-dependent and dose-dependent manners. However, copper transporter 1 was previously reported to be essential for MAPK signal transduction induced by FGF, PDGF, and EGF [24]. A potential explanation is that the major copper influx transporter, CTR1, maintains copper-dependent enzyme SOD1 which serves to inhibit phosphatases that limit RTK signaling, thus activating the central elements of RTK downstream signaling pathways. It has been reported that copper transporters and copper chaperones play essential roles in cardiovascular physiology and disease, including cell growth, migration, angiogenesis, and wound repair [25, 26]. Our data propose a new hypothesis that copper (II) might directly activate RTK signaling probably via the enhanced dimerization between monomer RTKs, which needs ultimate validation. Moreover, in addition to Ras/MAPK, and PI3K/PKB, the signal pathways mediated by RTK also include JNK, P38 MAPK, Rac, and the JAK/STAT pathway [1]. There are potential effects of copper (II) on other downstream signaling pathways of RTK activation. Finally, copper was previously demonstrated to be necessary for carcinogenic BRAF signals and tumorigenesis via the binding-enhanced kinase activity of copper (I) on intracellular MAPK [27]. Therefore, whether copper (II) can stimulate other signaling pathways to affect the cancer cell behavior remains to be further studied. In our research, we clearly evidenced that copper (II) promotes the phosphorylation of RTK as well as essential intracellular AKT and ERK pathways in two different cancer cells, i.e., A549 and DU145, supporting the hypothesis that the presence of high amounts of copper (II) in a tumor microenvironment may promote the cancer cell proliferation even if the natural ligands are deficient [28]. Thus, our results suggest that copper (II) significantly induces ligand-independent RTK signal pathways and promotes both cell migration and proliferation of malignant cells, which provides useful data for the further study of the mechanism of the effect of copper (II) on cancer cells. Thus, the understanding of role of copper (II) in cancer cell behaviors would contribute to the careful clinical preevaluation on not only the detectable ligands for RTK but also the concentration of copper (II) in the tumor microenvironment. Nevertheless, developing additive or synergistic treatment of copper (II) chelation combined with RTK inhibitors may lead to a potential survival advantage in cancer treatment.

## 5. Conclusion

Our results demonstrated that copper (II) ions could induce ligand-independent RTK-mediated signaling, promoting cell proliferation and wound healing. Copper (II) ions are bioactive ions and are crucial in the regulation of cell signaling pathway and cellular behaviors.

## Data Availability

The data used to support the findings of this study are available from the corresponding author upon request.

## References

[1] Schlessinger J. (2000). Cell signaling by receptor tyrosine kinases. *Cell*.

[2] Zhu W., Qin L. (2017). GOLM1-regulated EGFR/RTK recycling is a novel target for combating HCC metastasis. *Science China Life Sciences*.

[3] Liu Q., Zhang J., Gao H. (2018). Role of EGFL7/EGFR-signaling pathway in migration and invasion of growth hormone-producing pituitary adenomas. *Science China Life Sciences*.

[4] Zinkle A., Mohammadi M. (2018). A threshold model for receptor tyrosine kinase signaling specificity and cell fate determination. *F1000Research*.

[5] Östman A., Böhmer F.-D. (2001). Regulation of receptor tyrosine kinase signaling by protein tyrosine phosphatases. *Trends in Cell Biology*.

[6] Haj F. G., Markova B., Klaman L. D., Bohmer F. D., Neel B. G. (2003). Regulation of receptor tyrosine kinase signaling by protein tyrosine phosphatase-1B. *The Journal of Biological Chemistry*.

[7] Regad T. (2015). Targeting RTK signaling pathways in cancer. *Cancers*.

[8] Barberán S., Cebrià F. (2019). The role of the EGFR signaling pathway in stem cell differentiation during planarian regeneration and homeostasis. *Seminars in Cell & Developmental Biology*.

[9] Lv N., Hao S., Luo C. (2018). miR-137 inhibits melanoma cell proliferation through downregulation of GLO1. *Science China Life Sciences*.

[10] Skead G., Govender D. (2015). Gene of the month: MET. *Journal of Clinical Pathology*.

[11] Fresno Vara J. A., Casado E., de Castro J., Cejas P., Belda-Iniesta C., González-Barón M. (2004). PI3K/Akt signalling pathway and cancer. *Cancer Treatment Reviews*.

[12] Tang F., Pacheco M. T. F., Chen P., Liang D., Li W. (2018). Secretogranin III promotes angiogenesis through MEK/ERK signaling pathway. *Biochemical and Biophysical Research Communications*.

[13] Festa R. A., Thiele D. J. (2011). Copper: An essential metal in biology. *Current Biology*.

[14] Kaplan J. H., Maryon E. B. (2016). How mammalian cells acquire copper: an essential but potentially toxic metal. *Biophysical Journal*.

[15] Ding P., Zhang X., Jin S. (2017). CD147 functions as the signaling receptor for extracellular divalent copper in hepatocellular carcinoma cells. *Oncotarget *.

[16] Hu Frisk J. M., Kjellén L., Kaler S. G., Pejler G., Öhrvik H. (2017). Copper regulates maturation and expression of an MITF: tryptase axis in mast cells. *The Journal of Immunology*.

[17] Gopal A., Kant V., Gopalakrishnan A., Tandan S. K., Kumar D. (2014). Chitosan-based copper nanocomposite accelerates healing in excision wound model in rats. *European Journal of Pharmacology*.

[18] Kornblatt A. P., Nicoletti V. G., Travaglia A. (2016). The neglected role of copper ions in wound healing. *Journal of Inorganic Biochemistry*.

[19] Weichselbaum L., Klein O. D. (2018). The intestinal epithelial response to damage. *Science China Life Sciences*.

[20] Xie H., Kang Y. J. (2009). Role of copper in angiogenesis and its medicinal implications. *Current Medicinal Chemistry*.

[21] Shi X., Stoj C., Romeo A., Kosman D. J., Zhu Z. (2003). Fre1p Cu2+ reduction and Fet3p Cu1+ oxidation modulate copper toxicity in saccharomyces cerevisiae. *The Journal of Biological Chemistry*.

[22] Closson K. R., Paul E. A. (2014). Comparison of the toxicity of two chelated copper algaecides and copper sulfate to non-target fish. *Bulletin of Environmental Contamination and Toxicology*.

[23] Mandinov L., Mandinova A., Kyurkchiev S. (2003). Copper chelation represses the vascular response to injury. *Proceedings of the National Acadamy of Sciences of the United States of America*.

[24] Tsai C.-Y., Finley J. C., Ali S. S., Patel H. H., Howell S. B. (2012). Copper influx transporter 1 is required for FGF, PDGF and EGF-induced MAPK signaling. *Biochemical Pharmacology*.

[25] Urso E., Maffia M. (2015). Behind the link between copper and angiogenesis: established mechanisms and an overview on the role of vascular copper transport systems. *Journal of Vascular Research*.

[26] Fukai T., Ushio-Fukai M., Kaplan J. H. (2018). Copper transporters and copper chaperones: Roles in cardiovascular physiology and disease. *American Journal of Physiology-Cell Physiology*.

[27] Brady D. C., Crowe M. S., Turski M. L. (2014). Copper is required for oncogenic BRAF signalling and tumorigenesis. *Nature*.

[28] Brady D. C., Crowe M. S., Greenberg D. N., Counter C. M. (2017). Copper chelation inhibits BRAFV600E-driven melanomagenesis and counters resistance to BRAFV600E and MEK1/2 inhibitors. *Cancer Research*.

